# Genotype-specific responses of maize plants to *Funneliformis mosseae* under drought stress: phenomic and transcriptomic insights

**DOI:** 10.3389/fpls.2025.1723031

**Published:** 2026-01-06

**Authors:** Eszter Virág, Zoltán Zombori, Miklós Hóvári, Géza Hegedűs, László Sass, Györgyi Ferenc, Dénes Dudits, Katalin Posta

**Affiliations:** 1 Department of Planetary Health, Institute of One Health, Faculty of Health Science, University of Debrecen, Debrecen, Hungary; 2 Research Institute for Medicinal Plants and Herbs Ltd., Budakalász, Hungary; 3 Institute of Plant Biology, Hungarian Research Network (HUN-REN) Biological Research Centre, Szeged, Hungary; 4 Department of Information Technology and Its Applications, Faculty of Information Technology, University of Pannonia, Zalaegerszeg, Hungary; 5 Department of Microbiology and Applied Biotechnology, Institute of Genetics and Biotechnology, Hungarian University of Agriculture and Life Sciences, Gödöllő, Hungary

**Keywords:** arbuscular mycorrhizae, drought tolerance, genotype specificity, phenomics, transcriptomics

## Abstract

**Introduction:**

Drought is a major abiotic constraint limiting maize productivity, yet the genotype-specific mechanisms through which arbuscular mycorrhizal fungi (AMF) enhance drought resilience remain poorly understood. This study aimed to elucidate how AMF modulate drought tolerance, root plasticity, and heterosis in maize genotypes with contrasting drought sensitivity.

**Methods:**

Two maize inbred lines differing in drought tolerance (K1, tolerant; K2, sensitive) and their hybrid (KH) were grown under controlled pot conditions at either well-watered (60% soil moisture) or drought-stressed (30% soil moisture) levels, with or without inoculation with *Funneliformis mosseae* (BEG12). Integrated phenomic, biomass, and transcriptomic analyses were performed to characterize genotype-specific AMF responses.

**Results:**

AMF induced distinct, genotype-dependent responses under drought stress. In K1 plants, AMF maintained drought tolerance by stabilizing photosynthetic performance, supported by sustained expression of PSI, PSII, LHCb, and Calvin-cycle genes, alongside the activation of CYP450 71A1 and CONSTANS-like 3, suggesting auxin-associated regulation of stress adaptation. In contrast, drought strongly suppressed photosynthetic gene expression in K2 plants, while AMF promoted pronounced root system expansion accompanied by the induction of indole-3-acetaldehyde oxidase, auxin-binding protein 1, CORONATINE-INSENSITIVE 1, and tasselseed-2, indicating hormone-driven root plasticity and modified reproductive signaling. In the hybrid KH, selective activation of RbcX and heterosis-associated genes supported biomass stability and consistent flowering, although AMF had limited effects on hybrid vigor.

**Discussion:**

These findings reveal distinct molecular strategies underlying AMF-mediated drought resilience in maize, demonstrating that drought-sensitive genotypes primarily benefit through enhanced root plasticity, whereas drought-tolerant genotypes maintain photosynthetic stability. Overall, the results highlight the potential of targeted AMF–genotype combinations to improve water-use efficiency and promote sustainable maize production under drought stress.

## 1 Introduction

Drought is one of the most critical abiotic constraints limiting global maize productivity, affecting water-use efficiency, nutrient uptake, and overall soil–plant function. Given the increasing frequency of water scarcity, improving drought resilience has become a central challenge in sustainable maize production and soil health management. Current strategies for mitigating drought stress include the deployment of hybrid maize genotypes that exploit heterosis and the use of beneficial soil microorganisms, such as arbuscular mycorrhizal fungi (AMF) (Bruce et al., 2002; Boomsma and Vyn, 2008). Although hybrid vigor is widely utilized in global maize production, significant progress has been made in uncovering the genetic and physiological basis of heterosis (Wu et al., 2021; Pitz et al., 2025). The increased vigor of hybrids can be expressed throughout the life cycle of plants, as in early green biomass production, plant height (Shi et al., 2023), root system (Baldauf and Hochholdinger, 2023), early maize ear inflorescence formation (Ding et al., 2014), embryo development (Djisbar and Gardner, 1989), and seed yield (Bhatla et al., 2025).

Transcriptomic analyses of elite hybrids have revealed extensive non-additive gene expression patterns that influence carbon metabolism, glycolysis, and stress responses (Ding et al., 2014; Wan et al., 2022). Under drought conditions, the contribution of overdominant and underdominant genes to heterosis becomes particularly pronounced, influencing phenylalanine metabolism, carbohydrate turnover, glucose pathways, and thermotolerance (Sang et al., 2022).

In parallel with genetic approaches, AMF symbiosis represents a powerful ecological strategy for enhancing drought tolerance by improving root vigor, water and nutrient acquisition, and metabolic resilience (Ma et al., 2022). AMF can increase phosphorus and nitrogen availability, modulate soil organic carbon stability (Ma et al., 2025), and influence the temporal dynamics of plant responses to drought (Agnolucci et al., 2019, 2020; Sbrana et al., 2022). Genotype-specific differences in AMF responsiveness are well documented; for example, B73 relies heavily on AMF-mediated phosphorus uptake, whereas Mo17 upregulates its own phosphate transporter genes (Giovannini et al., 2022). Additional AMF-induced benefits include enhanced nitrogen uptake via AMF-inducible, ammonium transporters (Hui et al., 2022). Modulation of defense enzyme expression (Mayer et al., 2019) and improved resistance to potassium deficiency (Xu et al., 2024). Recent studies have also emphasized that *F. mosseae*-mediated drought tolerance is a multicomponent process (van Der Heijden et al., 2015) and that AMF effects vary across plant developmental stages (Abrar et al., 2024; Khan et al., 2024). Posta and Duc (2020) outlined the direct benefits of AM symbiosis in host plants under water deficit. These authors uncovered functional diversity among seven AMF species. The mechanisms induced by *F. mosseae* colonization have also been described in tomato plants under drought and heat stress (Duc et al., 2023; Szentpéteri et al., 2024). Moreover, root-associated microbiomes can influence the manifestation of heterosis in maize (Wagner et al., 2021), highlighting the importance of microbial interactions in shaping genotype performance.

Despite these advances, a critical knowledge gap remains: it is not yet understood how AMF colonization differentially modulates drought tolerance in maize inbred lines and hybrids, nor how the underlying phenomic and transcriptomic mechanisms vary in a genotype-dependent manner. In particular, the extent to which AMF enhances root plasticity in drought-sensitive genotypes, stabilizes photosynthetic processes in drought-tolerant genotypes, or alters heterosis in hybrids under water-limited conditions, remains unclear.

To address these gaps, we aimed to disentangle the genotype-specific effects of *F. mosseae* colonization on drought tolerance in maize using integrated phenomic, biomass, and transcriptomic analyses. We hypothesize that (i) AMF colonization differentially enhances drought tolerance among genotypes; (ii) drought-sensitive genotypes primarily benefit from AMF-induced root plasticity, whereas drought-tolerant genotypes rely on AMF-mediated stabilization of photosynthetic and metabolic processes; and (iii) hybrids exhibit distinct AMF-responsive phenomic and gene expression patterns that diverge from parental lines. By linking whole-plant phenotypes with molecular responses, this study advances the mechanistic understanding of AMF × genotype interactions and provides actionable insights into targeted symbiosis-based strategies to improve drought resilience in maize production systems.

## 2 Materials and methods

### 2.1 Plant material and growth conditions

The experiment was conducted in the greenhouse of the HUN-REN Biological Research Centre, Szeged, Hungary (46°14’44.0”N 20°09’54.8”E), from March to May 2023. For this study, hybrid maize seeds (referred to as KH) and seeds of its parental inbred lines (referred to as K1 (tolerant) and K2 (sensitive) plants) were used. The seeds were provided by the Kiskun Kutatóközpont breeding company (Kiskun Kutatóközpont Ltd., Kiskunhalas, Hungary). Seeds were pre-germinated for two days, and eight germinated seeds from each genotype and each treatment were planted into radio-tagged Plexiglass columns (400 mm height × 90 mm diameter)surrounded by polyvinyl chloride tubing. The columns were filled with a sterilized mixture of 80% peat soil (Florimo, Kecel, Hungary) and 20% sand, supplemented with 6 g Osmocote (Substral, Evergreen Garden Care, UK) as a nutrient source. The dry weight of the soil mixture was 1,050 ± 5 g in each column. The columns were filled up to approximately 75% of capacity, and then the AMF-containing culture was mixed into the last quartile. For non-mycorrhizal plants, a sterilized inoculum of the same quantity was mixed into the soil. The columns were placed in random order within an automatic modular phenotyping system (Photon System Instruments, Drasov, Czech Republic) The plants were watered to maintain 60% field capacity (soil water, MC) for control conditions and 30% MC for drought stress (Shi et al., 2023). Irrigation was performed automatically by the phenotyping system. The growth temperature was maintained at 24°C during the day and 20°C at night, with relative humidity ranging between 60% and 80% during the 6 weeks of cultivation. The plants were exposed to 14 h of light. Illumination levels were measured at approximately 400 μmol photons m^−2^ s^−1^ during the day, with minor daily variations detected throughout the experiment. A representative figure of plants in the phenotyping system is presented in **Supplementary Figure 1**.

### 2.2 Mycorrhizal inoculant

Isolates of *F. mosseae* BEG 12 (formerly *Glomus mosseae*) were propagated for 6 months in sterilized sand using *Zea mays* L. var. saccharata and *Medicago truncatula* L. were grown together as host plants under controlled greenhouse conditions. Throughout the experiment, plants were maintained at a 25 ± 2 °C/18 ± 2 °C day–night temperature regime, 50%–60% relative humidity, and a 16 h light/8 h dark photoperiod with a minimum photosynthetic photon flux density (PPFD) of 250 µmol m^−2^ s^−1^–300 µmol m^−2^ s^−1^. The inoculant acquired in this manner contained mycelia, spores, colonized root fragments of *F. mosseae*, and substrate (sterilized sand).

Fifty grams of inoculum substrate containing 30 spores g^−1^ were used for mycorrhizal inoculation of each plant, while plants without inoculation were supplemented with 50 g of autoclaved (three-times-sterilized at 121 °C for 60 min) AMF inocula (BEG 12) and an aliquot (3 ml) of a filtrate (<20 μm) of inocula to homogenize microbial populations without mycorrhizal propagules.

### 2.3 Assessment of mycorrhizal colonization of AM fungi

Mycorrhizal colonization was estimated before harvesting by visually inspecting fungal structures after clearing the roots in 10% KOH and staining with a vinegar–ink solution, as described by Vierheilig et al. (1998). The percentage of mycorrhizal colonization was calculated according to the gridline intersect method by observing 50 root pieces under a stereomicroscope (Giovannetti and Mosse, 1980).

### 2.4 Digital imaging of shoot and root development

Irrigation and digital imaging of the maize plants were performed twice a week. Water consumption was recorded, and irrigation was automatically managed using the phenotyping system. For shoot photography, the columns were automatically conveyed into an imaging chamber equipped with a rotatable platform, where the maize shoots were photographed using two RGB digital cameras (Cseri et al., 2013). The cameras captured seven images from the side and one from the top. The software automatically segments the images and calculates the plant parameters based on predefined specifications. For root density predictions using digital imaging, the plexiglass columns were photographed from four different side positions and from the bottom using two Canon EOS 600D digital cameras. Root-related white pixels were identified by subtracting the black soil background from the images to characterize the root area appearing on the surface of the chamber. We present the root pixel values from the final data collection. MPH% was calculated using the formula MPH% = [H − (P1 + P2)/2]/(P1 + P2)/2 ∗ 100 formula.

### 2.5 RNA isolation and sequencing library construction

The samples for RNA isolation were collected from the leaves of maize plants at identical developmental stages and immediately frozen in liquid nitrogen. The samples were ground in a mortar into a fine powder, and approximately 50 µg of this material was used for total RNA isolation. The procedure was carried out using the Quick-RNATM Plant Miniprep Kit (Zymo Research, Seattle, WA, USA) following the manufacturer’s instructions.

The concentration of the isolated total RNA was measured using the Equalbit BR RNA Assay kit (Vazyme Biotech). RNA Integrity Numbers were determined using a Labchip GX Touch Nucleic Acid Analyzer (Revvity) on a DNA 5K/RNA/CZE chip (Revvity) with an RNA Assay Reagent kit (Revvity). For Gene Expression Profiling (GEx) library construction, the QuantSeq 3′ mRNA-Seq Library Prep Kit FWD for Illumina (Lexogen) was used according to the manufacturer’s protocol. The library quantities were measured using the Equalbit 1× dsDNA HS Assay kit (Vazyme Biotech) on an Infinite Pro 200 F Nano+ Fluorescent Plate Reader (Tecan). The fragment size distribution of the libraries was determined by capillary electrophoresis on Labchip GX Touch Nucleic Acid Analyzer (Revvity) on an XMark HT chip (CLS144006, Revvity) using a DNA NGS 3k Assay kit (CLS960013, Revvity). Libraries were diluted to 700 pM for 1 × 75 bp single-end (22–24 million reads per library) sequencing with a 100-cycle 1.5B Reagent Kit on the NovaSeq X Plus Sequencing System (Illumina) according to the manufacturer’s protocol. The sequencing libraries were deposited in the NCBI Sequence Read Archive (SRA) under Bioproject PRJNA1267826.

### 2.6 Preprocessing of RNA-Seq reads

The preprocessing step involved quality control (QC), trimming, and filtering of raw fastq files. QC analysis was conducted using FastQC software v0.12.1 (Andrews, 2010), and Phred-like quality scores (Q scores) were set to >30. Low-quality reads, reads with adapter sequences at the ends, and reads exhibiting terminal skewing were removed using Trimmomatic software (Bolger et al., 2014). Contaminant sequences and reads containing ambiguous bases (Ns) were filtered out using the custom-developed application GenoUtils, as previously described (Mátyás et al., 2019). Reads with more than 30% Ns were discarded and reads with a lower N content were trimmed to a final length of >65 base pairs.

### 2.7 Mapping of RNA-Seq reads to the reference genome

The processed reads were mapped to the *Zea mays* reference genome (GCF_902167145.1), which was obtained from the NCBI on 13 January 2025. Bowtie2 was used for read alignment. Reads that matched the reference genome coding sequences (CDS) were retained for further analysis. The CDS sequences were reannotated, including Blastx alignment, Gene Ontology (GO) mapping, GO annotation, and functional analysis (GO-Slim). Blast alignments were performed against the NR database (version dated 13 January 2025), utilizing taxonomic filters for *Viridiplantae*, *Z. mays* (NCBI:txid33090), and *Z. mays* (NCBI:txid4577). This mapping was automated using OmicsBox (Bioinformatics Made Easy, BioBam Bioinformatics, https://www.biobam.com/omicsbox, accessed on 03.02.2025) (Götz et al., 2008).

### 2.8 Gene-level quantification and analysis of differential expressed genes

Sequencing reads were aligned to quantify gene expression from the RNA-Seq data, and the results were annotated in relation to genomic features. A count table (abundance matrix) was constructed for CDS sequences to facilitate downstream analysis. To identify genes with significantly different expression levels between experimental groups, pairwise differential expression analysis was performed using DESeq, a Bioconductor package (version 3.20). Three biological replicates were included for each condition in the study. Top50 pairwise DEGs are displayed in a heatmap indicating hierarchical clustering based on the Euclidean distance between genes. Expression data were determined using the logarithm of the CPM. This differential expression analysis was combined with an enrichment analysis of the examined sample pairs. The Generalized Linear Model (GLM) quasi-likelihood F-test was applied in the analysis of DEGs. Pairwise comparisons were designed to dissect the individual and combined effects of *F. mosseae* colonization and water availability on gene expression across three genetic backgrounds: K1, the drought-tolerant parental genotype; K2, the drought-sensitive parental genotype; and KH, the K1 × K2 hybrid genotype. Differentially expressed genes (DEGs) were identified under both control (60% MC) and drought (30% MC) conditions by comparing mycorrhizal (M+) and non-mycorrhizal (M−) plants within the same genotype and treatment groups. To assess the effect of *F. mosseae* colonization under well-watered (60% MC) and drought (30% MC) conditions, the following comparisons were made: (i) K1MC vs. K1C and K1MD vs. K1D; (ii) K2MC vs. K2C and K2MD vs. K2D; and (iii) KHMC vs. KHC and KHMD vs. KHD. A detailed comparison is provided in **Table 1**. These comparisons allowed the identification of AMF-responsive genes in a genotype- and water regime-specific manner. This design also enabled the evaluation of genotype-dependent responses to both drought and *F. moseae* symbiosis. Together, these pairings provide a comprehensive view of the transcriptional reprogramming influenced by AMF colonization under varying environmental conditions.

**Table 1:** Experimental sample-pair design used for differential gene expression (DEG) analysis across three maize genotypes (K1, K2, and KH) under well-watered and drought conditions, with or without *F. mosseae* colonization.

Genotype	Water condition	Mycorrhiza status	Comparison (M+ vs. M–)	Purpose
K1	60% MC (Control)	M+ vs. M–	K1MC vs. K1C	Effect of AMF under optimal water supply in drought-tolerant parental plants
	30% MC (Drought)	M+ vs. M–	K1MD vs. K1D	Effect of AMF under drought in drought-tolerant parental plants
K2	60% MC (Control)	M+ vs. M–	K2MC vs. K2C	Effect of AMF under optimal water supply in drought-sensitive parental plants
	30% MC (Drought)	M+ vs. M–	K2MD vs. K2D	Effect of AMF under drought in drought-sensitive parental plants
KH: K1 × K2 Hybrid	60% MC (Control)	M+ vs. M–	KHMC vs. KHC	Effect of AMF under optimal water supply in hybrid plants
	30% MC (Drought)	M+ vs. M–	KHMD vs. KHD	Effect of AMF under drought in hybrid plants

K1, drought-tolerant parental inbred line; K2, drought-sensitive parental inbred line; KH, K1×K2 hybrid. Water conditions: 60% MC, well-watered control; 30% MC, drought. Mycorrhiza status: M+, *F. mosseae* colonized plants; M−, non-colonized plants. Sample pair comparisons (M+ vs. M−) were used to evaluate the effect of AMF under each water condition and genotype. MC, moisture content; K1MC, *F. mosseae* colonized K1 plants well-watered control; K2MC, *F. mosseae* colonized K2 plants well-watered control; KHMC, *F. mosseae* colonized KH plants well-watered control; K1D, *F. mosseae* colonized K1 plants drought; K2D, *F. mosseae* colonized K2 plants drought; KHD, *F. mosseae* colonized KH plants drought.

### 2.9 Gene set enrichment analysis

GSEA was performed as described by Subramanian et al. (2005) to analyze pairwise sample comparisons. Gene ontology (GO) enrichment analysis was performed using GSEA to identify significantly enriched functional categories among differentially expressed genes. GO annotations were categorized into three main domains: Biological Process (BP), Molecular Function (MF), and Cellular Component (CC).

The pre-ranked GSEA method was applied using the fgsea algorithm which is part of the Bioconductor framework (version 3.21). Genes were ranked based on their differential expression statistics (log_2_ fold change), and enrichment scores (ES) were computed for each GO term using 1,000 permutations of gene labels. The enrichment score (ES) reflects the degree to which a GO term is overrepresented at the top or bottom of a ranked list. The raw ES values were adjusted to account for the size of the gene set to allow comparison across gene sets of different sizes (NES), which corrects for bias because larger gene sets tend to have higher raw ES simply due to their size.

GSEA results were evaluated using the Normalized Enrichment Score (NES). For the analysis of the ranked gene list of DEGs, the following formula was applied:


$$Rank=sign(logFC)\cdot (log_{10}(p-Value))$$

where logFC represents the log2-fold change in expression between conditions.

The false discovery rate (FDR q-value) was calculated for correct multiple hypothesis testing. GO terms with FDR q <0.25 were considered significantly enriched in accordance with GSEA standards. The nominal p-value is also reported as a measure of statistical significance before correction (p <0.05). Analyses were performed separately for the BP, MF, and CC categories.

### 2.10 Pathway analysis

Pathway analysis was conducted using the Plant Reactome database (https://www.gramene.org/pathways) to provide insights into the underlying biological mechanisms associated with pairwise DEGs (Naithani et al., 2016). Annotated sequences from the enriched DEGs (derived from GSEA) were linked to relevant biological pathways. Gene products were matched with the most likely candidates found in the pathway database through an intermediate step. This mapping was automated using OmicsBox (Bioinformatics Made Easy, BioBam Bioinformatics, https://www.biobam.com/omicsbox, accessed on 03.02.2025).

### 2.11 Determination of RPM digital gene expression units for genes of interest

Based on the results of the pathway and GSEA analyses, genes from differentially altered and functionally defined groups (genes of interest, GOIs) were selected. The Reads Per Million mapped reads (RPM) values for these GOIs were determined and compared across samples. The GOIs were filtered from five functional categories of *Z. mays* genes that exhibited changes across all samples: auxin, jasmonate, and brassinosteroid signaling and indole-3-acetic acid (IAA) biosynthesis. RPM values for flowering-related genes were calculated by selecting subsets of GOIs. RPM is a simple and effective normalization method for digital gene expression analysis, and is especially suitable for single-end 75 bp Illumina reads with approximately 20 million reads per sample. It accounts for differences in sequencing depth by scaling raw read counts to per-million mapped reads, allowing for direct comparisons across samples.

### 2.12 Statistical analysis

All phenotypic measurements (shoot biomass, plant height, root pixel area, water consumption, and water-use efficiency (WUE)) were analyzed using one-way or two-way analysis of variance (ANOVA), depending on the experimental design (genotype × water regime × mycorrhiza). Statistical significance was set at p <0.05. Data are presented as mean ± standard deviation (SD). For image-derived root traits, pixel-based root area values were subjected to the same ANOVA framework with the same *post hoc* criteria. Correlation analyses between phenotypic and physiological parameters were performed using Pearson’s correlation coefficient, with significance accepted at p <0.05. RNA sequencing data were analyzed using the DESeq pipeline, where DEGs were defined as FDR <0.05 (Benjamini–Hochberg correction). GO and GSEA were evaluated based on NES, nominal p <0.05, and FDR q <0.25, according to the standard GSEA criteria.

## 3 Results

### 3.1 *F. mosseae* colonization levels and water used efficiency across genotypes and moisture conditions

**Figure 1:**
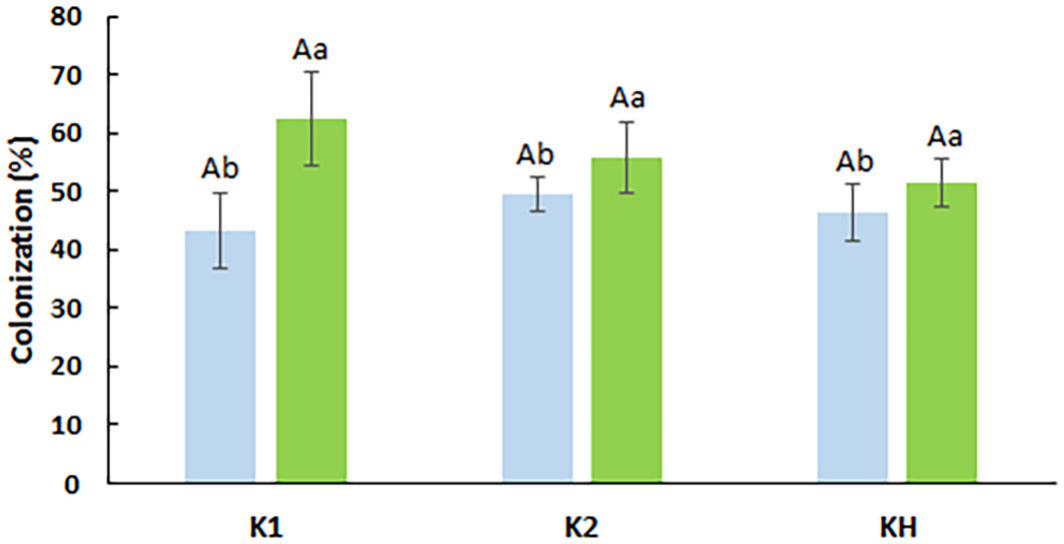
*F. mosseae* colonization rates in maize genotypes. K1, drought-tolerant parental genotype; K2, drought-sensitive parental genotype; KH, K1 × K2 hybrid. Different capital letters indicate significant differences among the genotypes under the same conditions (60% MC—blue; 30% MC—green) according to Games–Howell *post hoc* test (p <0.05). Different regular letters indicate significant differences among the same genotypes under different conditions according to Tukey’s *post hoc* test (p <0.05).

The level of AMF colonization varied across maize genotypes under different water supply conditions. Plants of all genotypes exhibited higher colonization under limited water supply (30% MC). The K1 inbred plants showed the highest drought-stimulated colonization (**Figure 1**). In the KH: K1 × K2 hybrid line, AMF colonization was intermediate under both water conditions.

**Figure 2:**
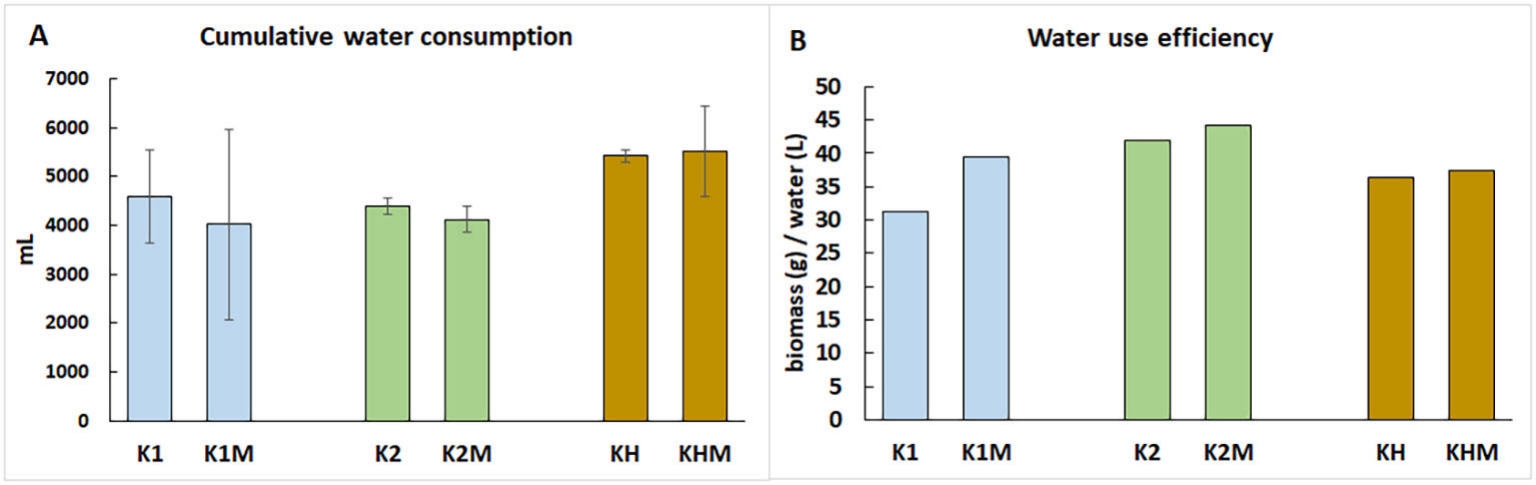
Genotype-dependent water consumption **(A)** and WUE **(B)** under drought stress and normal water supply with or without mycorrhiza symbiosis. K1, drought-tolerant parental genotype; K2, drought-sensitive parental genotype; KH, K1 × K2 hybrid, C, 60% watering; D, 30% watering; M, mycorrhizal inoculation.

Cumulative water consumption (**Figure 2A**) did not differ substantially between the K1 and K2 plants under 60% MC. Under 30% MC, K1 plants used more water than did drought-sensitive K2 plants. AMF marginally affected water uptake. KH plants maintained adequate water use under all treatments, and AMF did not alter their water consumption.

Water-use efficiency (WUE) (**Figure 2B**) was strongly dependent on the genotype. K1 plants showed higher WUE under drought than 60% MC, with little effect of AMF. KH plants also exhibited stable WUE across all treatments. In K2 plants, AMF dramatically increased WUE under drought conditions, resulting in a sevenfold improvement, indicating a substantial symbiotic benefit in this genotype.

### 3.2 Shoot growth heterosis under drought and *F. mosseae* colonization

**Figure 3:**
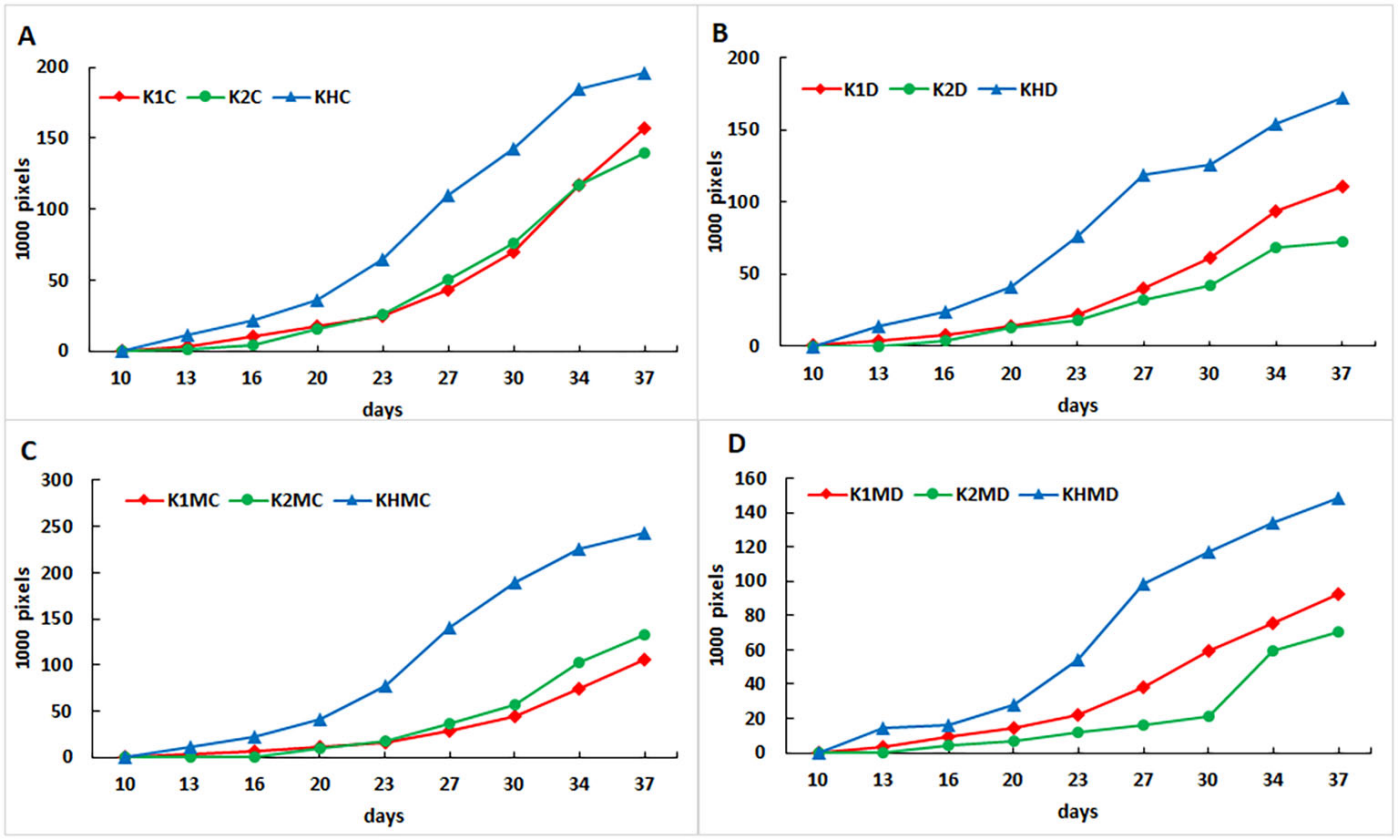
Biomass of shoot growth dynamics represented by the above-ground green pixel number of maize plants from the two parental lines and their crossing hybrid under the different treatments over the time of the experiment. **(A)** 60% FC; **(B)** Drought, 30% FC; **(C)** 60% MC with *F*. *mosseae* inoculation; **(D)** drought, 30% MC content with mycorrhiza inoculation. Legends: K1, drought-tolerant parental genotype; K2, drought-sensitive parental genotype; KH, K1 × K2 hybrid.

Early indicators of hybrid vigor include seedling growth rate and biomass formation, which can be effectively quantified using high-throughput phenotyping (HTP) platforms under controlled conditions (Kim et al., 2020). In this study, the crossing combination examined displayed clear hybrid advantages. At ≤60% MC, HTP pixel data showed that the hybrid had higher biomass than both inbred lines from the second week onward (**Figure 3A**), and this difference continued to increase. The final biomass measurements reflected the same trend (**Table 2**), with an MPH of 20.18%.

**Table 2:** Plant height, shoot fresh weight, and mid-parent heterosis (MPH%) of three maize genotypes (K1, K2, and KH) under well-watered (60% MC) and drought (30% MC) conditions, with and without *F. mosseae* colonization.

Mycorrhiza status	Watering	Genotype	Height (cm)	MPH%	Fresh weight (g)	MPH%
M-	60% MC	K1	117.2 ± 5.2	12.1 ± 1.9	143.2 ± 14.2	20.2 ± 3.2
		KH	142.7 ± 2.3		197.1 ± 6.2	
		K2	137.4 ± 5.5		184.8 ± 5.7	
	30% MC	K1	86.2 ± 10.0	84.1 ± 8.3	99.6 ± 11.7	238.6 ± 32.9
		KH	138.9 ± 4.6		188.6 ± 11.8	
		K2	64.7 ± 2.1		11.8 ± 1.8	
M+	60% MC	K1	105.7 ± 8.2	29.8 ± 6.5	159.1 ± 8.0 *	20.6 ± 6.1
		KH	144.1 ± 6.9		205.9 ± 9.8	
		K2	116.3 ± 6.2		182.3 ± 33.0	
	30% FC	K1	82.8 ± 8.2	61.3 ± 8.9	93.3 ± 7.9	91.8 ± 16.8
		KH	135.0 ± 6.5		193.2 ± 8.4	
		K2	84.6 ± 8.2 ***		108.2 ± 31.5***	

K1, drought-tolerant parental inbred line; K2, drought-sensitive parental inbred line; KH, K1 × K2 hybrid. Water regimes: 60% MC, well-watered control; 30% MC, drought. Mycorrhiza status: “M+”, plants colonized by *F. mosseae*; “M−”, non-colonized plants. MPH% (mid-parent heterosis) was calculated as: MPH% = H−(P1 + P2)/2H − (P1 + P2)/2H − (P1 + P2)/2/(P1 + P2)/2(P1 + P2)/2(P1 + P2)/2 × 100.

Values represent mean ± SD; asterisks indicate statistically significant differences (p < 0.05).

Drought stress at 30% MC caused a substantial decrease in the green biomass pixel values in all genotypes (**Figure 3B**). By the end of the experiment, inbred K1 plants showed a biomass reduction of 43.6 g, K2 plants showed 173.0 g, whereas the hybrid exhibited only an 8.5 g decrease (**Table 2**). These results indicate that the hybrid tolerated water limitation far better, leading to an MPH value more than 10 times higher than that under optimal moisture conditions.

We also examined the influence of *F. mosseae* inoculation on shoot growth at both moisture levels (**Figure 3**; **Table 2**). Under 60% MC, pixel values indicated a stimulative effect of 26.2% in the hybrid, whereas K1 and K2 plants showed reduced pixel values (**Figures 3A, C**). Under 30% MC, pixel numbers were slightly lower in K1 plants and KH plants, whereas in K2 plants, no reduction was observed following colonization (**Figures 3B, D**). Final biomass measurements showed modest stimulation under 60% MC in K1 (15.9 g) and KH (8.8 g) plants, whereas K2 plants showed no increase. Under 30% MC, AMF increased shoot biomass in KH plants (4.7 g) and produced a substantial increase in drought-sensitive K2 plant biomass (96.4 g). AMF did not alter hybrid vigor (MPH) under optimal moisture, but under reduced soil moisture, the MPH value was lower than that in non-inoculated hybrid plants.

The plant height data revealed similar trends (**Table 2**). Drought reduced height by 30.9 cm in K1 plants, 4.2 cm in KH plants, and 72.7 cm in K2 plants. Mycorrhizal inoculation decreased plant height in both inbreds at 60% MC, whereas at 30% MC, it increased height only in the drought-sensitive K2 plants, which grew 20 cm taller than non-inoculated plants.

Sexual organ development was recorded at the end of the experiment as an additional phenotypic marker. All hybrid plants formed both ears and tassels in all treatments (**Supplementary Table 1**). AMF stimulated ear formation in K1 plants at both soil moisture levels, and tassel development was more advanced under 30% MC. In K2 plants, AMF delayed ear development at 60% MC, whereas tassel formation was enhanced under drought.

### 3.3 Root system phenotypes under optimal and drought conditions influenced by *F. mosseae*

**Figure 4:**
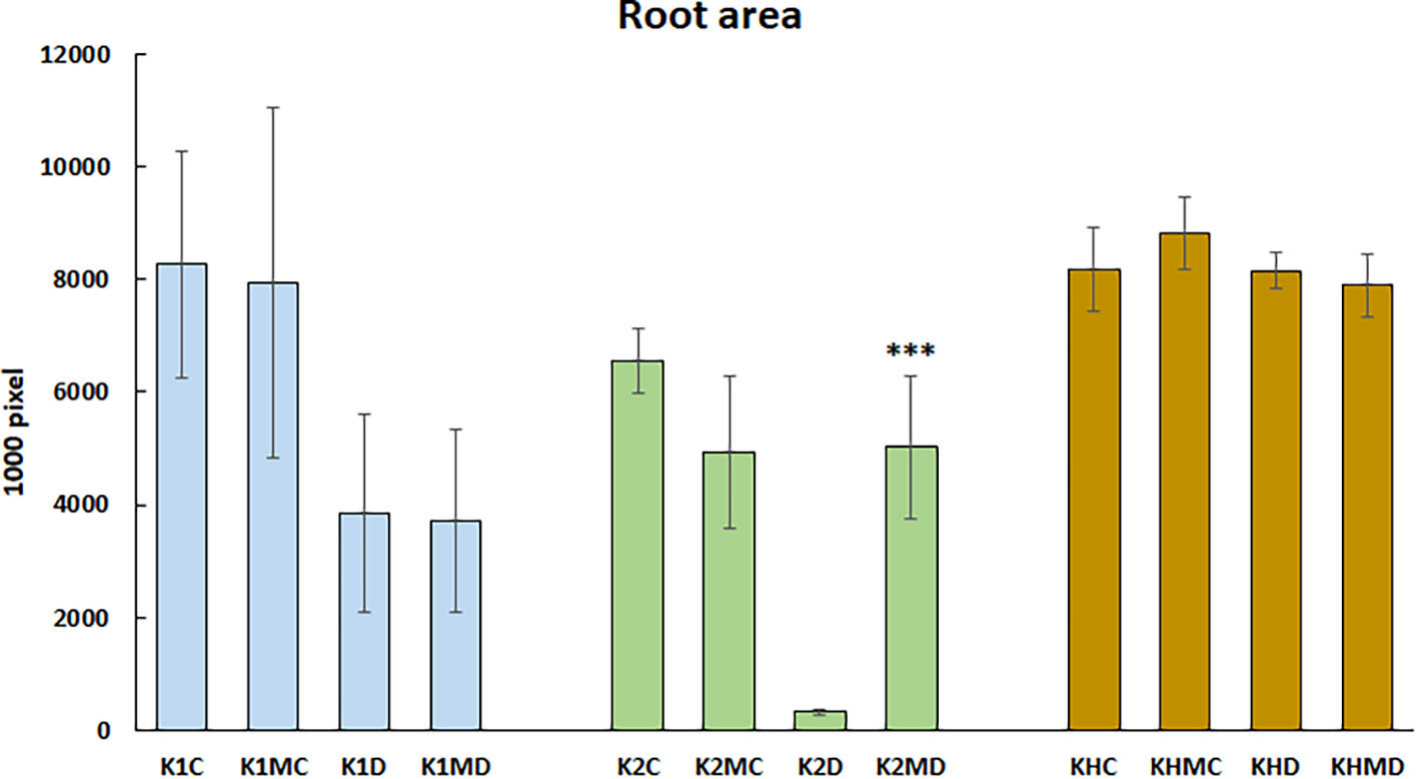
Root biomass represented by the white pixel number of the root system of the maize plants from the two parental lines and their crossing hybrid under the different treatments at the end of experiment. C, 60% FC; D, Drought, 30% FC; M, *F. mosseae* inoculation; K1, drought-tolerant parental genotype; K2, drought-sensitive parental genotype; KH, K1 × K2 hybrid. asterisks indicate statistically significant differences (p < 0.05).

AMF are beneficial soil microbes that directly colonize roots, thereby influencing root system development. In the present study, we monitored the root system structure using digital imaging. As shown in **Figure 4**, under optimal moisture (60% MC), the K2 inbred plants developed smaller root systems. Under drought (30% MC), strong genotype-dependent changes were observed: the root system of drought-sensitive K2 plants was almost completely diminished, K1 plants showed an approximately 50% reduction, whereas the KH hybrid maintained substantial root growth. Correspondingly, MPH increased from 10.2% ( ± 9.1) to 292.1% (± 47.9) under moisture limitation.

Regarding the effects of mycorrhizal inoculation, K2 plants showed the most distinct response (**Figure 4**). Under 60% MC, AMF colonization reduced root size, whereas under 30% MC, it markedly enhanced root development. Mycorrhizal inoculation increased heterosis under well-watered conditions, but under drought conditions, the MPH value was lower than at 60% MC. Specifically, MPH changed from 37.1% ( ± 12.6) under 60% MC to 80.5% ( ± 22.7) under moisture limitation.

### 3.4 Genotype-specific responses of DEGs to *F. mosseae* colonization under drought stress

**Figure 5:**
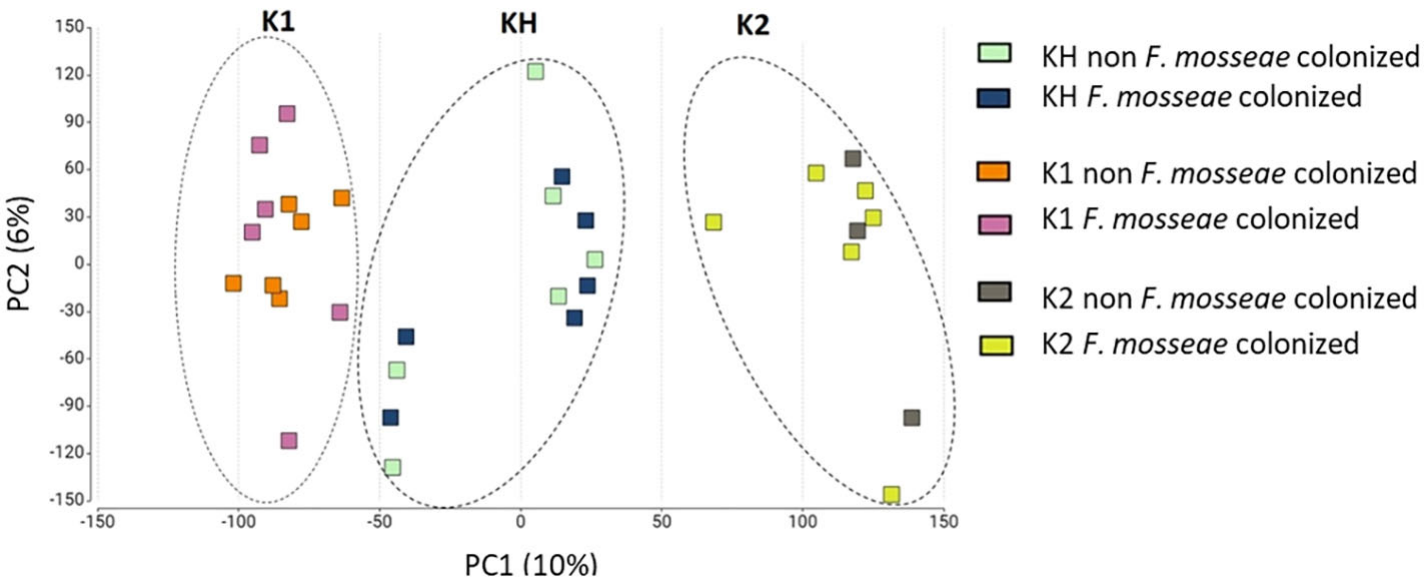
Two-dimension PCA plots of samples based on read abundances. The PCA analysis examined the following components: K1, drought-tolerant parental genotype; K2, drought-sensitive parental genotype; and KH, K1 × K2 hybrid plants. The X axis (PC1, 10%) explains the major variance and primarily separates the genotypes, while the Y axis (PC2, 6%) accounts for treatment-specific variance associated with *F. mosseae*-colonization and FC. Each point represents an individual biological replicate, with dashed ellipses indicating genotype-specific clusters.

To explore global transcriptional differences among the drought-tolerant K1 plants, drought-sensitive K2 plants, and KH hybrid plants under drought and *F. mosseae* colonization, we performed PCA on normalized gene expression data. The two-dimensional PCA plot (**Figure 5**) showed clear a separation of the three genotypes. PC1 mainly distinguished K1 plants from K2 plants, reflecting their contrasting drought tolerance, whereas PC2 separated the KH plants from both parental lines.

The hybrid plants occupied an intermediate position between their parents, indicating that their transcriptomic profile combined elements of both genotypes and reflected additive or partially dominant expression patterns. This intermediate clustering also suggests that KH plants undergo distinct but parentally influenced transcriptional adjustments in response to drought and AMF colonization. Overall, PCA demonstrated strong genotype-dependent divergence in transcriptomic responses, with the hybrid exhibiting a unique expression profile shaped by both parental backgrounds.

Venn diagrams (**Supplementary Figure 2**) showed that AMF inoculation increased the number of upregulated DEGs in K1 plants under drought (3,361 under drought compared to 1,732 under 60% MC), whereas the number of downregulated genes decreased (2,231 vs. 2,983). In K2 plants, AMF induced a large set of commonly upregulated genes (890), but the number of downregulated genes increased notably under drought conditions (2,525 vs. 1,198). In KH plants, AMF induced 2,284 and 1,419 upregulated genes under 60% and 30% MC, respectively, with drought causing extensive downregulation (5,562 vs. 1,619), highlighting major AMF-mediated transcriptomic reprogramming (**Table 3**).

**Table 3:** Summary of differentially expressed genes (DEGs) in mycorrhizal (M+) vs. non-mycorrhizal (M−) maize plants under well-watered (60% MC) and drought (30% MC) conditions across three genotypes (K1, K2, and KH).

Genotype (M+ vs. M−)	Condition	Upregulated (unique)	Upregulated (shared)	Downregulated (unique)	Downregulated (shared)	Key trend
K1	60% MC	1,732	362	2,983	323	Moderate activation, many downregulated genes
K1	30% MC	3,361	362	2,231	323	Stronger activation, fewer downregulated genes
K2	60% MC	2,460	890	1,198	1,998	Balanced activation, moderate repression
K2	30% MC	1201	890	2,525	1,998	Strong repression under drought
KH	60% MC	2284	218	1,619	451	Moderate activation, limited repression
KH	30% MC	1419	218	5,562	451	Extensive downregulation, distinct reprogramming

K1, drought-tolerant inbred line; K2, drought-sensitive inbred line; KH, K1 × K2 hybrid. Water regimes: 60% MC, well-watered; 30% MC, drought.

Mycorrhiza status: M+, *F. mosseae* colonized plants; M−, non-colonized plants.

“Unique”, DEGs occurring exclusively in the given condition; “Shared”, DEGs common with the other water condition of the same genotype.

Values represent total numbers of significantly up- or downregulated DEGs identified in M+ vs. M− comparisons.

### 3.5 GO enrichment highlights distinct *F. mosseae*-mediated drought adaptation strategies

**Figure 6:**
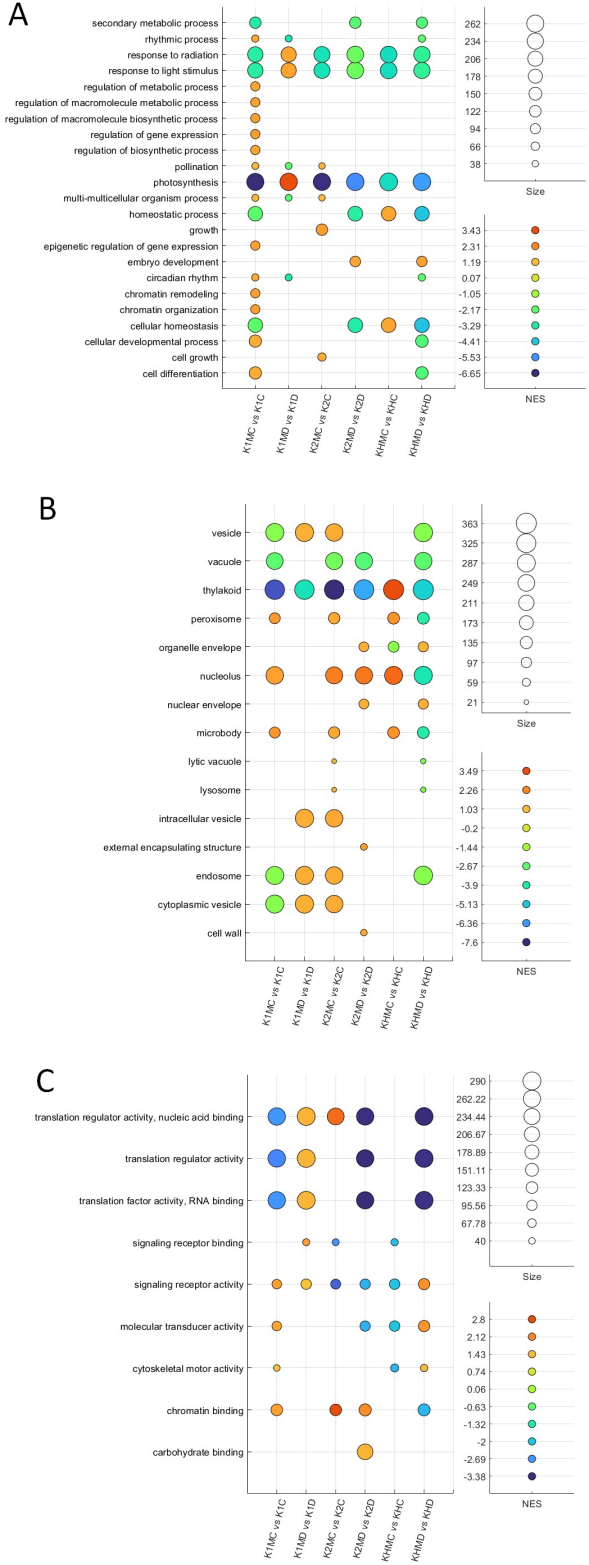
GO enrichment analysis of DEGs in maize genotypes colonized by *F*. *mosseae* under control (60% FC) and drought (30% FC) conditions. **(A)** Biological process categories show strong enrichment in secondary metabolism, photosynthesis, regulation of gene expression, and stress responses, with clear genotype-specific differences. **(B)** Cellular component categories highlight nucleus-, vacuole-, thylakoid-, and vesicle-associated DEGs, reflecting distinct subcellular regulation in K1 plants, K2 plants, and KH plants. **(C)** Molecular function categories are dominated by transcriptional and translational regulators, signaling receptor binding, and chromatin binding, particularly enriched in the KH hybrid. Bubble size indicates the number of DEGs, while color represents the normalized enrichment score (NES).

GO enrichment analysis revealed clear genotype- and treatment-dependent patterns across biological processes, cellular components, and molecular functions associated with *F. mosseae-*responsive DEGs (**Table 4**). Within the biological process category (**Figure 6A**), AMF colonization strongly affected genes involved in secondary metabolism, rhythmic processes, and responses to light and radiation, particularly in K2 and KH hybrid plants. Photosynthesis-related processes were enriched among the upregulated genes in drought-treated K1 plants, whereas this category was less pronounced in K2 plants. In KH plants, the enrichment of processes linked to gene expression regulation, chromatin organization, and development indicated substantial transcriptional reprogramming.

**Table 4:** Dominant Gene Ontology (GO) categories in three maize genotypes (K1, K2, and KH) under drought conditions, summarizing major biological processes, cellular components, and molecular functions associated with genotype-specific transcriptional responses.

Genotype	BP	CC	MF	Key trend
K1	Regulation of gene expression, chromatin organization, developmental processes	Nucleus, chromatin, nuclear envelope	Moderate enrichment of transcriptional regulators	Stable stress adaptation via transcriptional and epigenetic control
K2	Secondary metabolism, photosynthesis, light/radiation response	Thylakoid, peroxisome, vacuole	Translation regulator activity, RNA binding, transcription factor activity	Plastidial and metabolic adjustments, oxidative stress responses
KH	Secondary metabolism, photosynthesis, signaling-related processes	Vesicle trafficking, vacuole, cytoplasmic vesicle	Translation regulators, signaling receptor binding, molecular transducer activity	Broad transcriptional reprogramming with vesicle-mediated signaling and resource allocation

K1, drought-tolerant inbred line; K2, drought-sensitive inbred line; KH, K1 × K2 hybrid plants. GO classifications: Biological process (BP), major functional pathways activated under drought; Cellular component (CC), subcellular locations enriched among DEGs; Molecular function (MF), dominant molecular activities associated with enriched genes. Key trend summarizes the characteristic transcriptional response strategy of each genotype.

In the cellular component category (**Figure 6B**), the most enriched terms were vesicles, vacuoles, thylakoids, peroxisomes, and nuclei. Under drought conditions, KH plants exhibited the strongest enrichment of vesicle- and vacuole-associated DEGs, suggesting enhanced vesicle trafficking and vacuolar activity during stress. K1 plants showed enrichment of nucleus- and chromatin-related genes, whereas K2 plants were enriched in thylakoid and peroxisome components, consistent with plastidial and oxidative stress-related responses.

Within the molecular function category (**Figure 6C**), translation regulation and transcription factor activity dominated, with strong enrichment in both K2 and KH plants. The hybrid also showed increased signaling receptor binding and molecular transducer activity, reflecting intensified regulatory interactions during AMF colonization. In contrast, K1 plants showed moderate enrichment of transcriptional regulators, consistent with their stable stress-adaptive profile.

Overall, the GO results indicate distinct AMF-mediated functional responses: K1 plants rely mainly on transcriptional and chromatin-level regulation, K2 plants exhibit pronounced plastidial and metabolic adjustments, while KH plants undergo broad transcriptional reprogramming accompanied by enhanced vesicle-mediated and signaling processes.

Statistical tables including the Nominal P-value and FDR q-value of GO-categories are presented in **Supplementary Tables 2–4**.

### 3.6 Pathway analysis of *F. mosseae*-induced modulation in maize genotypes

**Figure 7:**
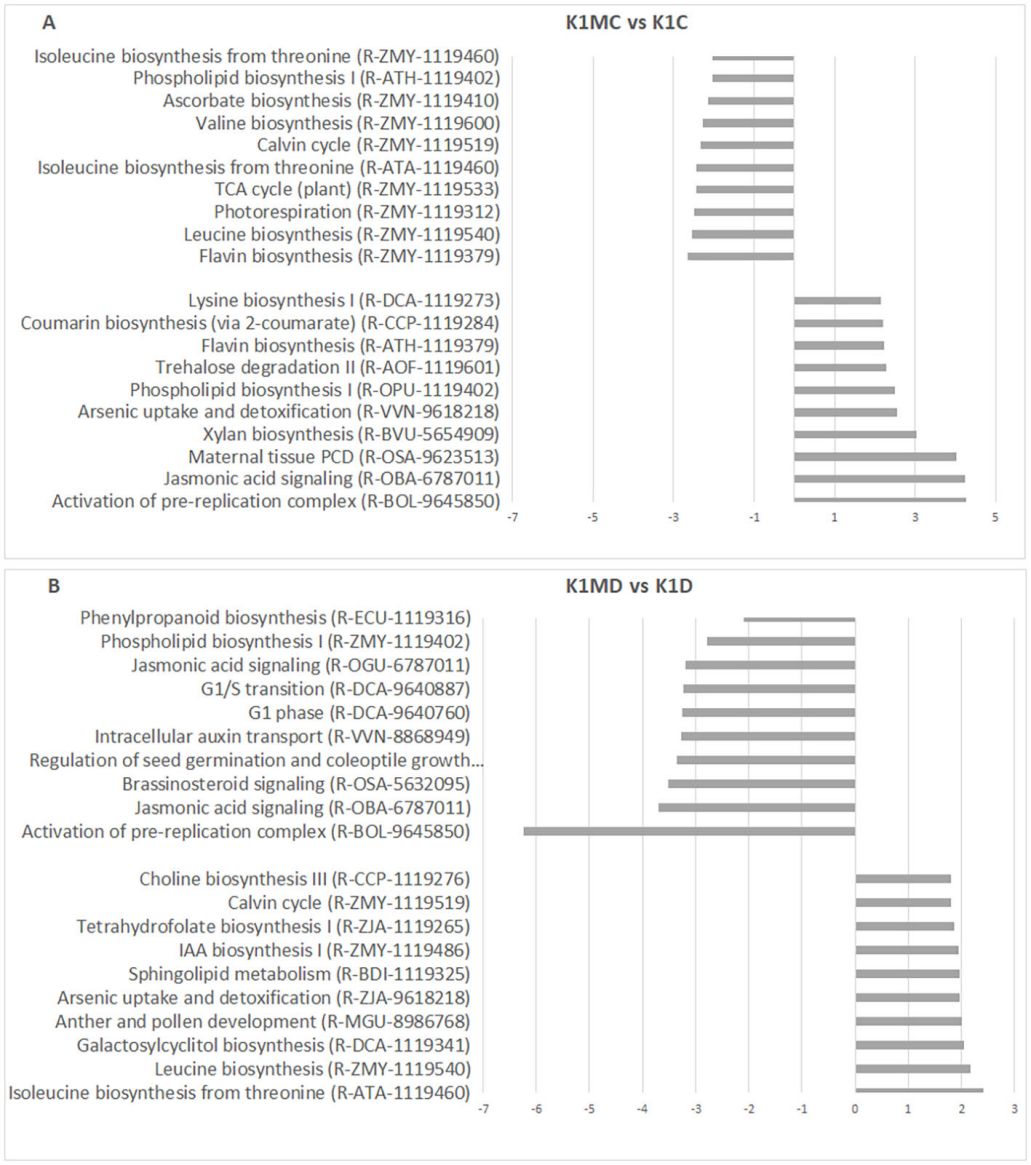
Analysis of enriched biochemical pathways was performed using the Plant Reactome Database. DEGs of the investigated sample pairs were used to categorize the Top 10 pathways showing the most substantial alterations based on the NES values (−3 or +3). Top 10 enriched pathways of sample pairs K1MC vs K1C **(A)** and K1MD vs K1D **(B)**. Categorization was performed according to NES values of gene sets aligned to the given pathways. K1, drought-tolerant parental genotypes; C, 60% MC without *F*. *mosseae* inoculation; D, Drought, 30% MC without *F*. *mosseae* inoculation; MC, 60% MC with *F*. *mosseae* inoculation; MD, Drought, 30% MC with *F*. *mosseae* inoculation.

In addition to the physiological responses described above, *F. mosseae* colonization influences several biochemical pathways. Therefore, we examined the ten most enriched pathways in each genotype under optimal (60% MC) and reduced moisture (30% MC) conditions. In the drought-tolerant K1 genotype, *F. mosseae* colonization under 60% MC caused a moderate increase in biomass (16 g, **Table 2**) and activated pathways related to the pre-replication complex, jasmonic acid (JA) signaling, programmed cell death in maternal seed tissues, and xylan biosynthesis (**Figure 7A**). Under drought conditions (30% MC), K1 plants did not show positive NES values, and only the pre-replication complex displayed negative enrichment (**Figure 7B**).

**Figure 8:**
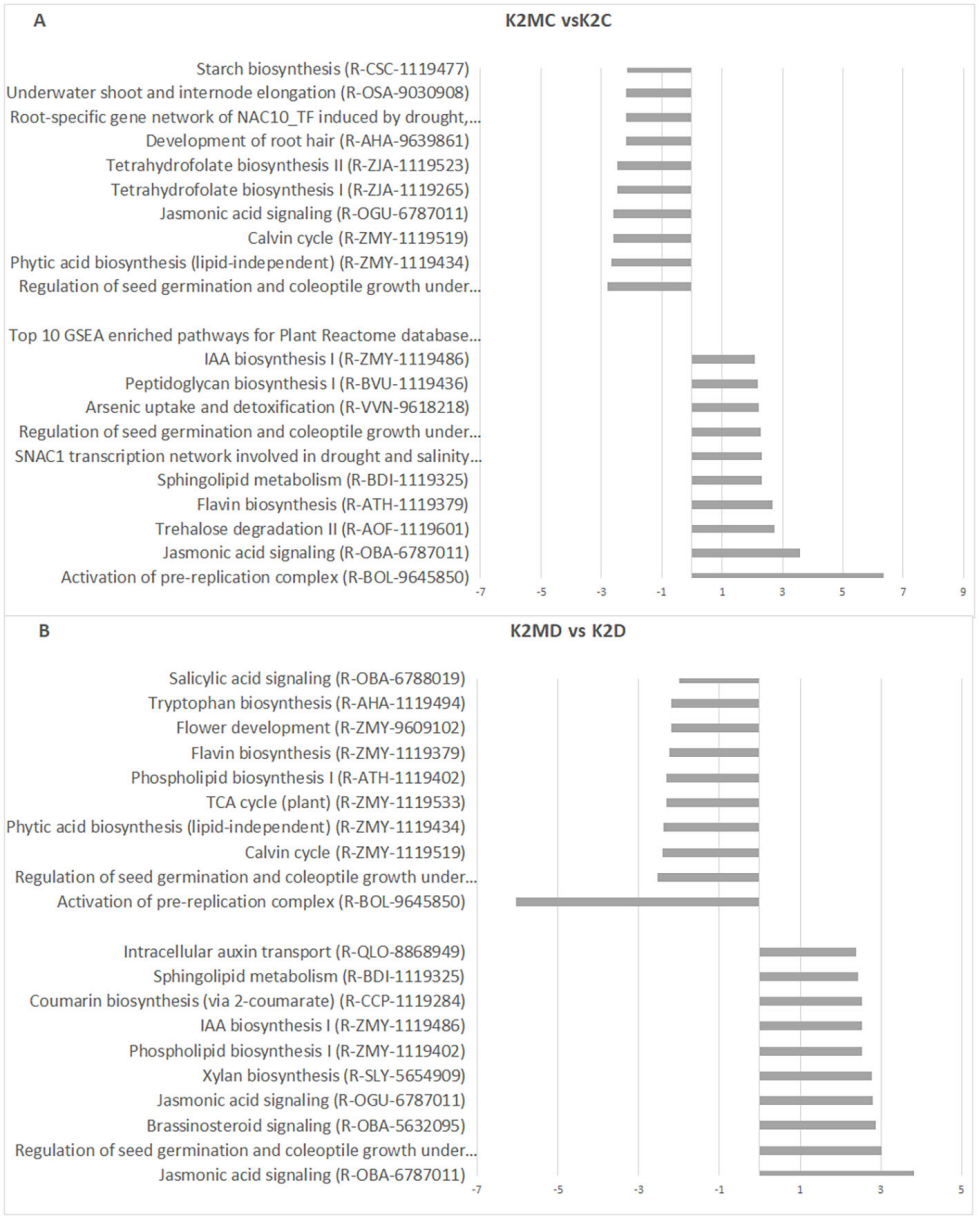
Top 10 enriched pathways of sample pairs K2MC vs K2C **(A)** and K2MD vs K2D **(B)**. Categorization was performed according to NES values of gene sets aligned to the pathways. K2, drought-sensitive parental genotypes; C, 60% MC without AMF inoculation; D, Drought, 30% MC without AMF inoculation; MC, 60% MC with AMF inoculation; MD, Drought, 30% MC with AMF inoculation.

In the drought-sensitive K2 genotype, *F. mosseae* activated the pre-replication complex and JA signaling under 60% MC (**Figure 8A**). Under 30% MC, JA signaling, seed germination, brassinosteroid (BR) signaling, and xylan biosynthesis were among the most strongly enriched pathways (**Figure 8B**), consistent with the pronounced biomass stimulation observed in this genotype (**Table 2**).

**Figure 9:**
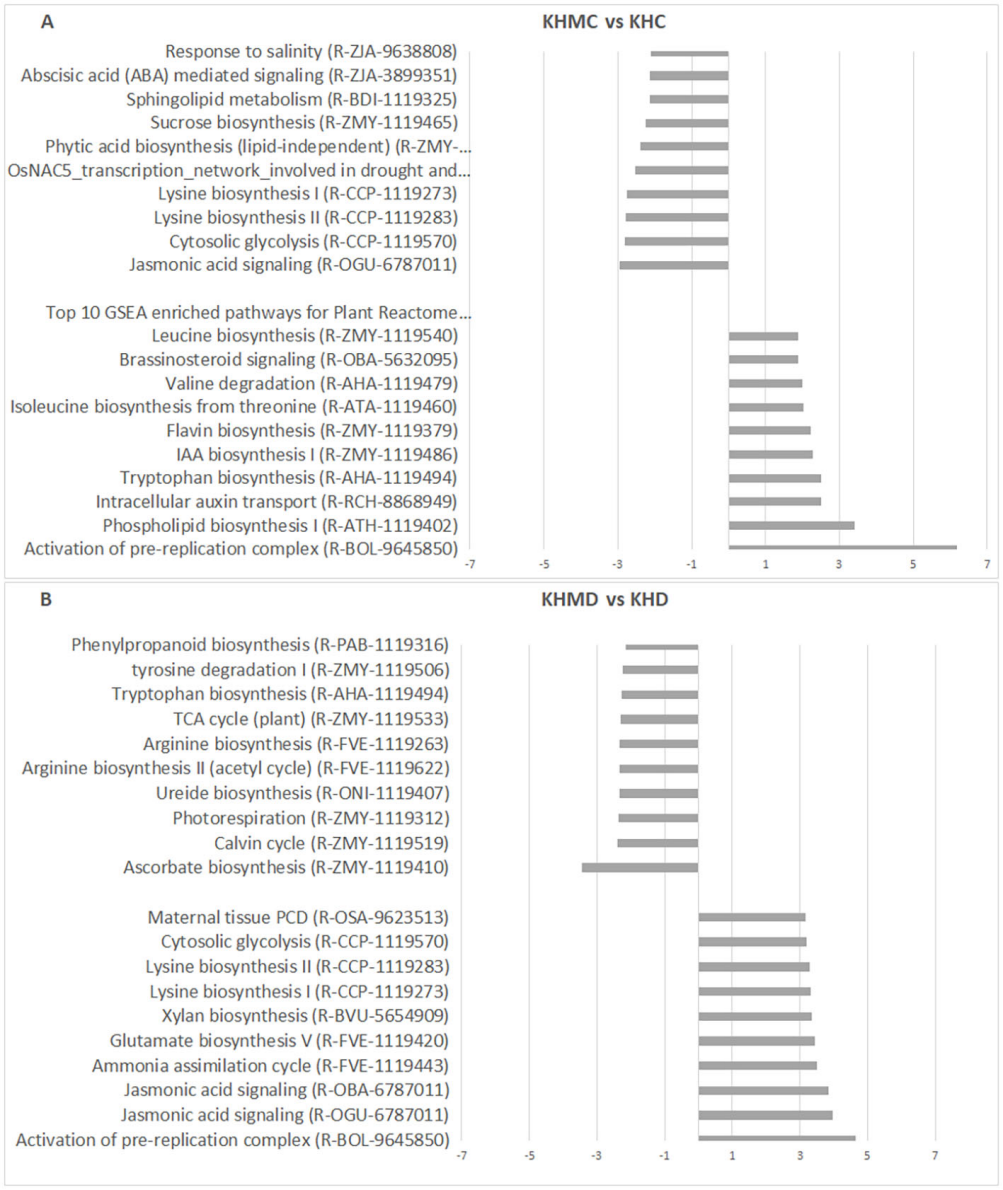
Top 10 enriched pathways of sample pairs KHMC vs KHC **(A)** and KHMD vs KHD **(B)**. Categorization was performed according to NES values of gene sets aligned to the pathways. KH, K1 × K2 hybrid; C, 60% MC without AMF inoculation; **(D)**, Drought, 30% MC without AMF inoculation; MC, 60% MC with AMF inoculation; MD, Drought, 30% MC with AMF inoculation.

In the KH (K1 × K2) hybrid plants, *F. mosseae* reduced JA signaling and cytoplasmic glycolysis under 60% MC while activating the pre-replication complex and phospholipid synthesis pathways. Under drought conditions (30% MC), ascorbate biosynthesis decreased, whereas multiple metabolic and signaling pathways, including the pre-replication complex, JA signaling, ammonia assimilation, glutamate biosynthesis, and xylan biosynthesis, were activated (**Figures 9A, B**).

Overall, JA pathway modulation by *F. mosseae* was clearly genotype-dependent. JA signaling was induced under 60% MC in both parental genotypes (K1 and K2), whereas under 30% MC, it was activated in K2 and KH plants but not in K1 plants. BR signaling also showed genotype-specific patterns; it was downregulated in K1 plants (**Figure 7B**) and upregulated in K2 plants under drought (**Figure 8B**), indicating its contribution to differential adaptation strategies.

The pre-replication complex pathway was activated by AMF under 60% MC in both K1 and K2 plants suggesting enhanced cell division under favorable moisture conditions. Under drought conditions (30% MC), this pathway was underrepresented in both parental lines but not in the KH hybrid, which maintained drought tolerance. These findings indicate that *F. mosseae* modulates cell cycle-related processes in a genotype- and soil moisture-dependent manner.

### 3.7 RPM analysis of photosynthesis-, hormone-, and reproduction-related DEGs under *F. mosseae* colonization

RPM profiling of DEGs across photosynthesis-, hormone-, and flowering-related pathways revealed clear genotype-dependent transcriptional adjustments to *F. mosseae* colonization. Complete RPM datasets for photosynthesis (238 genes), hormonal pathways (167 genes), and flowering genes (80 genes) are available in the Mendeley repository (DOI: 10.17632/6fc357zrbj.1), while ≥3-fold changes are shown in **Supplementary Figures 3, 4**.

For photosynthesis-related genes (**Supplementary Figure 3**), K1 plants maintained PSI, PSII, LHCB, and Calvin cycle gene expression under drought (30% MC) when colonized by *F. mosseae*, indicating stable photosynthetic performance. Examples include increases in transcripts encoding photosystem II protein D2 (from 1.33 RPM to 10.00 RPM) and photosystem I subunit d1 (from 12.67 RPM to 51.33 RPM). K2 plants showed pronounced downregulation of photosynthetic genes under drought, with only partial compensation by AMF (e.g., chlorophyll a-b binding protein CP29.1 decreased from 270.33 RPM to 45.01 RPM). In KH, drought led to widespread repression of photosynthetic genes, but several key regulators were selectively upregulated, such as RbcX protein 2 (16.33 RPM to 46.67 RPM) and photosystem I P700 apoprotein A1-like (3.08 RPM to 52.33 RPM), reflecting a hybrid-specific compensatory mechanism in response to drought.

Hormone-related gene responses (**Supplementary Figure 4**) also differed significantly. JA signaling genes, such as pnFL-2 and coronatine-insensitive 1a-like, were strongly induced by AMF in K2 plants under drought, while in K1 plants AMF mainly modulated JA signaling under 60% MC. In KH, AMF consistently reduced LCR69 expression across all moisture regimes. BR pathway components showed genotype-specific modulation: K1 plants primarily displayed drought-associated induction (e.g., LOC100272702), whereas K2 plants accumulated several low-activity BR signaling genes. In KH and K2, AMF inhibited the genes encoding 14-3-3-like proteins and LCR69, indicating altered hormone-mediated regulation.

Auxin biosynthesis and transport genes also responded to *F. mosseae* colonization by the roots. K1 plants showed drought-induced increases in Cytochrome P450 71A1, which was further enhanced by colonization. In K2 plants, genes such as indole-3-acetaldehyde oxidase and pyruvate decarboxylase 2 were particularly active under drought conditions with *F. mosseae* colonization. KH plants exhibited increased expression of auxin-related regulators, including IAA4 and IAA5, and selective induction of auxin signaling elements (e.g., ARF22, ABP5, and SKU5-like oxidases).

Reproduction-related genes also showed moisture- and genotype-dependent shifts. In K1 plants, AMF supported spike and tassel development, whereas drought strongly induced CONSTANS-like 3. In K2 plants, spike development was delayed under 60% MC with AMF, whereas tassel formation was stimulated through the induction of CONSTANS-interacting protein 6. In KH plants, AMF supported embryo development under drought conditions, consistent with the upregulation of key reproductive regulators such as knotted1 induced 1 precursor, tasselseed-2, and MADS19.

Together, the RPM profiles demonstrate that *F. mosseae* reshapes photosynthetic, hormonal, and reproductive transcriptional programs in a strongly genotype- and moisture-dependent manner in the host plant.

## 4 Discussion

There is a broad consensus that the symbiotic relationship between AMF and host plants confers a range of mutual benefits (Bonfante and Genre, 2010), including enhanced nutrient uptake, stress tolerance, and biomass accumulation. In our previous study on maize, AMF inoculation significantly increased biomass under controlled conditions (Mayer et al., 2019), supporting this paradigm. The present study expands on these findings through a multifactorial experimental design that simultaneously evaluates phenotypic traits, such as biomass productivity, water availability, and *F. mosseae* colonization, and transcriptomic changes, allowing a comprehensive exploration of how AMF modulates the activity of gene networks associated with photosynthesis, hormonal regulation, and reproductive development under both control and dry conditions. Uniquely, this integrative approach helped reveal potential systemic transcriptional and metabolic signatures associated with genotype-specific drought responses of maize plants under mycorrhizal infection. We identified previously uncharacterized cross-talk between photosynthetic gene regulation and hormone signaling networks, particularly involving the remodeling of reproductive development under drought conditions.

This study elucidates how *F. mosseae* colonization and heterosis-driven transcriptional regulation jointly shape whole-plant adaptive strategies in maize through systemic adjustments in photosynthesis, hormonal networks, and developmental programs that underpin genotype-dependent drought responses and hybrid vigor.

### 4.1 Systemic effects of *F. mosseae* colonization in drought adaptation

In this study, we examined how *F. mosseae* colonization triggers whole-plant systemic adjustments—spanning photosynthetic regulation, hormonal signaling, and reproductive development—that collectively shape genotype-dependent drought adaptation in maize. The systemic influence of colonization on drought adaptation reveals distinct genotype-specific responses at both physiological and molecular levels. In the drought-tolerant genotype K1, AMF colonization supported photosynthetic resilience by stabilizing the expression of genes encoding PSI and PSII subunits, Rubisco, and chlorophyll-binding proteins. This molecular stabilization translated into moderated biomass loss and reproductive development maintenance under water deficit, consistent with previous reports on AMF-mediated photoprotection and stress mitigation (Bahadur et al., 2019; Begum et al., 2019; Mathur et al., 2019; Yooyongwech et al., 2023; Huang et al., 2024; Ahmed et al., 2025). Importantly, in K1 plants, AMF enhanced energy metabolism via the upregulation of enzymes such as pyruvate, phosphate dikinase, and ATP synthase. These findings suggest an improved cellular energy status under symbiotic conditions. Concurrently, genes encoding stress-response proteins, such as HDA101, VQ motif proteins, and ERF transcription factors, exhibited attenuated activation in the presence of AMF, indicating a more balanced stress signaling profile. This modulation likely contributes to a controlled defense response that prevents energy over-expenditure during drought. Furthermore, auxin-related gene expression (e.g., *IAA17*, *IAA5*, *IAA18*, and *ARF22*) was downregulated under drought + AMF conditions, consistent with the fine-tuning effect of AMF on hormone signaling pathways. Together, these expression patterns imply that in tolerant genotypes, such as K1 plants, AMF colonization reinforces pre-existing drought-adaptive mechanisms through a combined effect on photosynthesis, energy metabolism, and hormonal regulation.

In contrast, in the drought-sensitive maize genotype K2, *F. mosseae* colonization resulted in a disproportionate increase in biomass and root growth despite the repression of photosynthetic genes, suggesting a compensatory shift toward hormone-mediated adaptation through hormonal reprogramming involving elevated JA and BR signaling. This pattern is consistent with previous findings that AMF can enhance JA and BR signaling under abiotic stress (Mathur et al., 2019; Huang et al., 2024), thereby influencing root architecture and enhancing stress resilience. In addition, the observed delay in ear formation, alongside accelerated tassel development, aligns with hormonal rebalancing between the AUX and BR pathways, a hallmark of stress-induced reproductive plasticity (Begum et al., 2019). The upregulation of the JA receptor COI1 and AUX-related genes encoding indole-3-acetaldehyde oxidase and auxin-binding protein 1, coupled with the repression of flowering regulators such as *tasselseed-2*, suggests a strategic shift away from reproductive investment toward vegetative endurance. This hormonal trade-off exemplifies a plastic response, wherein AMF assists in stress survival without rescuing the core photosynthetic pathways. As previously reported, such compensatory hormone-driven adaptation reflects the flexible yet limited benefits of AMF in sensitive backgrounds (Begum et al., 2019; Mathur et al., 2019; Huang et al., 2024). These findings indicate that in sensitive genotypes, *F. mosseae* colonization promotes an alternative adaptive strategy focused on systemic hormonal reprogramming rather than stabilizing photosynthetic processes. This finding reinforces earlier reports that AMF can exert systemic hormonal effects independently of photosynthetic enhancement (Bahadur et al., 2019; Yooyongwech et al., 2023; Ahmed et al., 2025).

KH hybrid plants displayed an intermediate response pattern, characterized by robust drought tolerance largely independent of AMF colonization. While phenotypic changes were minimal, transcriptomic data revealed substantial reprogramming, particularly a selective ~3-fold induction of the Rubisco assembly chaperone *RbcX*. This gene is implicated in Rubisco biogenesis and carbon fixation under stress (Saschenbrecker et al., 2007; Kolesiński et al., 2011), and its induction under drought+AMF conditions likely supports biomass preservation. Notably, KH plants displayed subdued hormonal responses, with only mild induction of auxin-responsive elements (*ARF22*, *ABP5*, and *SKU5-like oxidase* genes) and minimal changes in JA or BR signaling. This observation parallels previous work, indicating that heterotic genotypes often exhibit attenuated responsiveness to symbiotic interactions due to already optimized physiological traits (Quiroga et al., 2017; Hu et al., 2020). This hormonal minimalism aligns with the hybrid’s consistent ear and tassel development across treatments, suggesting that heterosis confers reproductive robustness with a reduced dependency on hormonal reprogramming. Such buffering of flowering traits in hybrids has been noted in maize, where heterosis provides stability under abiotic stress (Wagner et al., 2021; Pitz et al., 2025). The hybrid’s stable reproductive development and reduced hormonal volatility imply a buffering effect provided by heterosis, which may reduce the dependency on microbial symbiosis for stress resilience.

Under control, in irrigated conditions, *F. mosseae* colonization continued to exert a significant transcriptomic influence on K1 plants, particularly by enhancing the expression of photosynthesis-related genes encoding PsbP, PSI/PSII subunits, Rubisco, and protochlorophyllide reductase. This biostimulant-like effect was especially pronounced in the hybrid (KH plants), supporting the idea that AMF can enhance photosynthetic efficiency, even in non-stressful environments. Auxin signaling pathways showed divergent expression patterns in different maize genotypes; genes such as *IAA30* and *IAA17* were upregulated, whereas others were suppressed, indicating a nuanced role for AMF in growth regulation. Development-related transcription factors, including *CONSTANS-like*, *KN1*, and *MADS-box* genes, were also stabilized or enhanced in AMF-colonized plants, particularly in the hybrid, which may underpin its superior growth performance.

### 4.2 Heterosis-associated transcriptional patterns in the KH hybrid maize plants

We investigated how KH hybrid plants exhibit heterosis-associated transcriptional patterns byintegrating enhanced activation of growth- and hormone-related pathways with the selective suppression of stress-responsive regulators, which together form a whole-plant regulatory framework underpinning their superior vigor and developmental stability.

KH hybrid plants displayed a distinct transcriptional signature that reflected heterosis-related regulation. Several gene families showed elevated expression in the hybrid compared to both parents, particularly under irrigated conditions. Members of the TIFY family (*TIFY3*, *TIFY6a*, *TIFY10b*, and *TIFY11e*) were generally more strongly induced, consistent with a reinforced jasmonate- and auxin-linked signaling background. In parallel, auxin- and development-associated regulators, including *IAA13*, *IAA30*, *IAA4*, *ARF*22, CONSTANS-like, and knotted1-like transcription factors, were significantly upregulated, indicating the robust activation of hormonal and developmental pathways. Genes implicated in sex determination and developmental reprogramming (*tasselseed-2*, *knotted1-induced precursor*, and *homeobox knotted-1-like 11*) also exhibited strong induction, reflecting the stimulation of reproductive and vegetative growth programs that are typical of heterotic genotypes. In addition, energy metabolism–related genes encoding ATP synthase D chain, cytochrome P450, and photosynthetic proteins were more prominently expressed in the hybrid, suggesting an enhanced cellular energy status under non-stress conditions. Simultaneously, certain negative regulators of stress signaling, including specific isoforms of HDA101, ERF096, Ninja family protein 5, and VQ-motif proteins, remained repressed in the hybrid background. This downregulation, together with the attenuation of some TIFY family members under specific conditions, indicates a selective reduction in the negative signaling inputs. Taken together, the KH hybrid plants combined the upregulation of developmental and auxin/light-signaling pathways with the suppression of stress-sensitive regulators. Such dual regulation provides a plausible mechanistic basis for heterosis, where enhanced growth and reproductive stability are achieved through reinforced activation of positive regulators and attenuation of inhibitory elements (Saschenbrecker et al., 2007; Kolesiński et al., 2011; Quiroga et al., 2017; Hu et al., 2020; Wagner et al., 2021; Pitz et al., 2025). These findings support the view that heterotic genotypes rely on integrated transcriptional buffering, which stabilizes reproductive development under stress while maintaining superior energy metabolism and hormonal control.

## 5 Conclusion

This study offers the first integrative phenomic, transcriptomic, and metabolomic assessment of how *F. mosseae* colonization influences drought responses in tolerant, sensitive, and hybrid maize genotypes. Our findings clearly demonstrate that the effects of AMF are not uniform; instead of acting as general enhancers of stress tolerance, AMF reshape drought adaptation through genotype-specific regulatory mechanisms. Across all genotypes, AMF strengthened the coordination of key hormonal pathways, especially AUX, JA, and BR signaling, which supported developmental reprogramming linked to flowering regulation and root–shoot carbon allocation. In KH hybrid plants, the enhanced activation of growth- and reproduction-associated transcriptional programs, together with the suppression of negative regulators, provides a mechanistic explanation for superior heterosis and biomass stability under water deficit.

The results of the present complex studies highlight the importance of the host genetic background in determining the outcome of AMF symbiosis and underscore the value of multi-omics integration for dissecting plant–microbe interactions. The recognized expression patterns imply that in tolerant genotypes, such as K1 plants, AMF colonization reinforces pre-existing drought-adaptive mechanisms through a combined effect on photosynthesis, energy metabolism, and hormonal regulation. By contrast, in the drought-sensitive maize genotype K2, *F. mosseae* colonization resulted in a disproportionate increase in biomass and root growth despite repression of photosynthetic genes, suggesting a compensatory shift toward hormone-mediated adaptation, through hormonal reprogramming involving elevated JA and BR signaling.

The practical implications are significant because the presented data emphasize the importance of AMF inoculation, preferentially for drought-sensitive plant genotypes.

## Data Availability

The datasets presented in this study can be found in online repositories. The names of the repository/repositories and accession number(s) can be found below: https://www.ncbi.nlm.nih.gov/, PRJNA1267826.
