# Supplementary material for: Genotype-specific responses of maize plants to Funneliformis mosseae under drought stress: phenomic and transcriptomic insights
Source: Front Plant Sci. 2026 Jan 6;16:1723031. doi: 10.3389/fpls.2025.1723031 (PMC12816378; doi:10.3389/fpls.2025.1723031)
Supplement: Supplementary file 1 [file DataSheet1.docx]

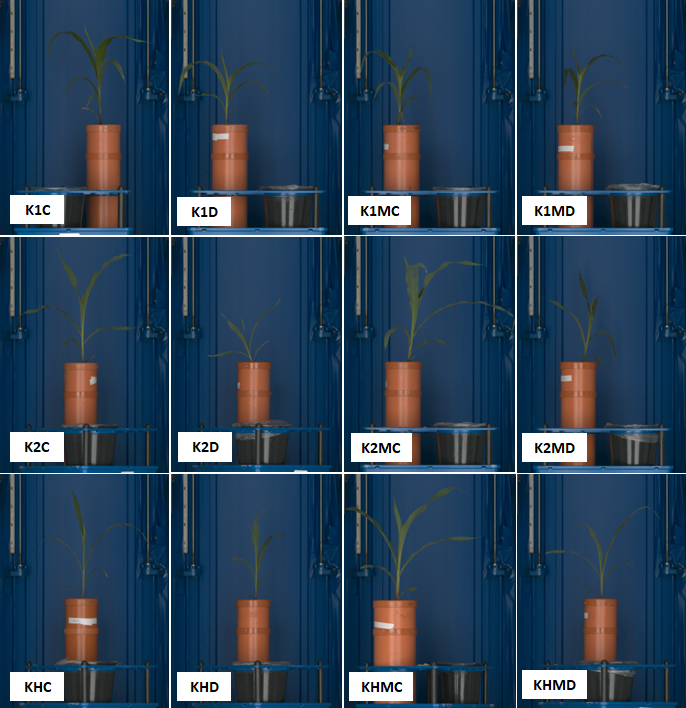


Supplementary Figure 1

Representative images of the 4 weeks old maize plants in the modular phenotyping system. K1: drought-tolerant parental genotype; K2: drought-sensitive parental genotype; KH: K1xK2 hybrid; C: 60% moisture content without *F. mosseae* inoculation, D: Drought, 30% moisture content without *F. mosseae* inoculation, MC: 60% moisture content with *F. mosseae* inoculation, MD: Drought, 30% moisture content with *F. mosseae* inoculation


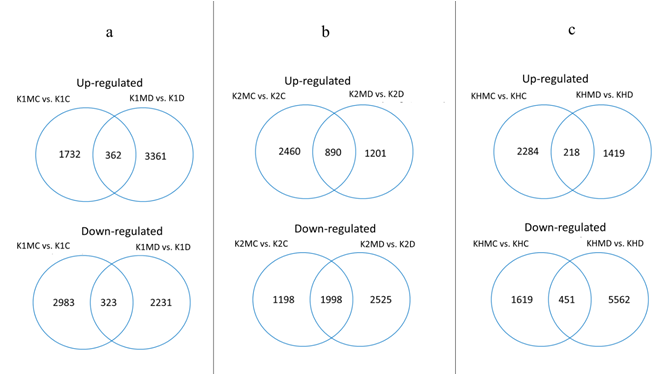


Supplementary Figure 2

Venn diagrams illustrating the number of differentially expressed genes (DEGs) that are up- or down-regulated in response to *F. mosseae*-colonization under different levels of drought stress in three maize genotypes. K1 (drought-tolerant) inbred line: comparison of AMF-inoculated and non-inoculated plants under moderate (60% FC, K1MC vs. K1C) and severe (30% FC, K1MD vs. K1D) water deficit (a). K2 (drought-sensitive) inbred line: a large number of shared up-regulated DEGs between moderate (60% FC) and severe drought (30% FC) conditions suggests a strong AMF-induced transcriptional response, indicating the critical role of *F. mosseae*-colonization in stress adaptation (b). KH: K1xK2 hybrid: fewer overlapping DEGs suggest a reduced reliance on AMF for drought response (c). These Venn diagrams quantify genotype-specific AMF effects on the transcriptome. The results highlight that in the K2 (drought-sensitive) genotype, AMF colonization is essential to activate drought-response mechanisms and ensure plant survival under water-limited conditions.

Supplementary Figure 3


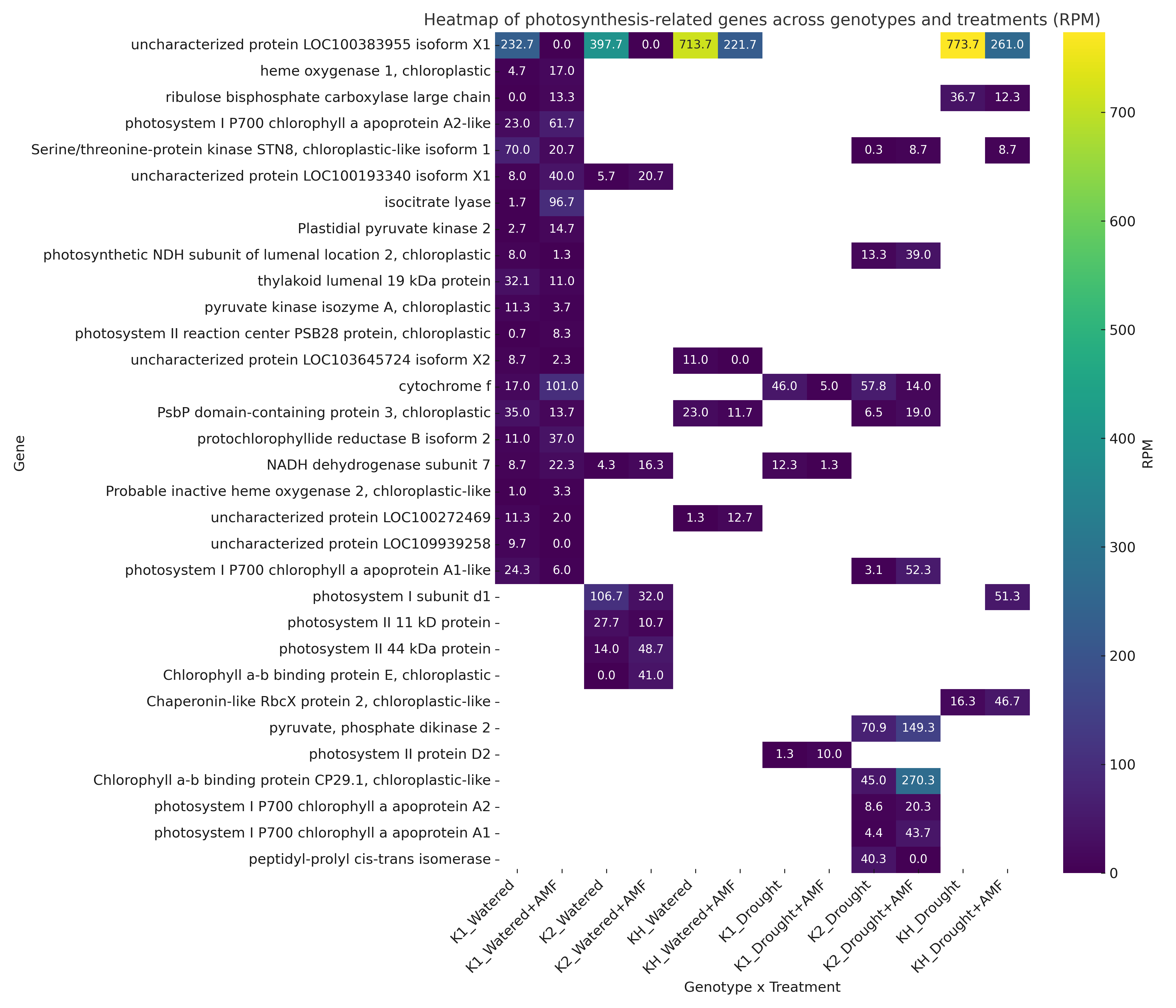


Heatmap representation of photosynthesis-related genes across genotypes (K1, K2, KH) and treatments (Watered, Watered+AMF, Drought, Drought+AMF). Values indicate normalized transcript abundance (RPM). Funneliformis mosseae colonization supported the stability of PSI, PSII and Calvin-cycle transcripts in drought-tolerant K1 plants, whereas drought-sensitive K2 plants exhibited strong repression (e.g., CP29.1, cytochrome f) with only partial AMF compensation. The KH hybrid displayed massive repression under drought but selective induction of key regulators (e.g., RbcX, PSI apoproteins), along with extremely high expression of uncharacterized LOC100383955, reflecting a compensatory stress response strategy.


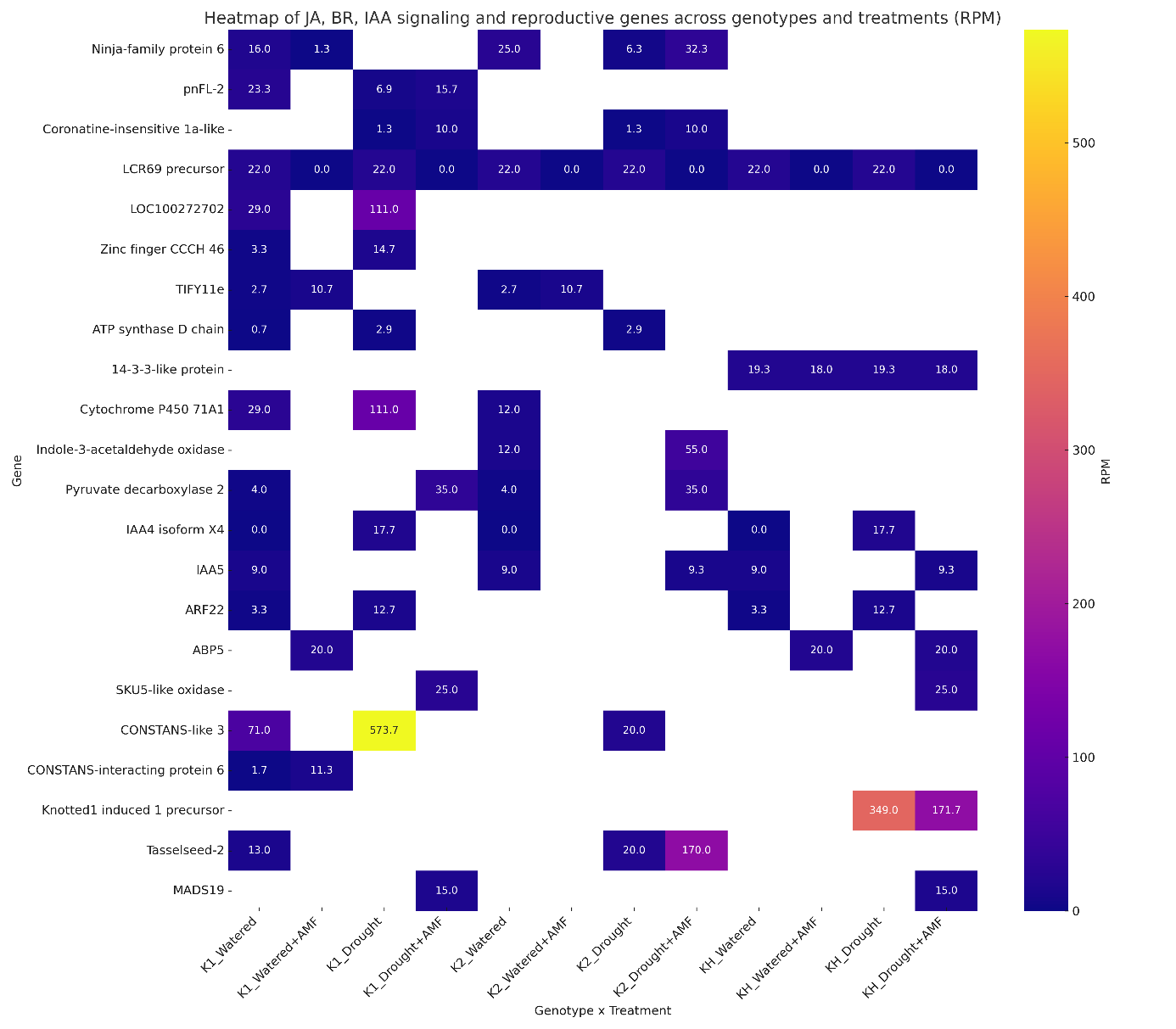


Supplementary Figure 4

Heatmap of JA, BR, IAA signaling and reproductive genes in K1, K2 and KH plants under four treatments (Watered, Watered+AMF, Drought, Drought+AMF). Transcript abundance is shown as reads per million (RPM). The heatmap illustrates AMF-mediated repression of ninja-family protein 6 in K1, induction of pnFL-2 under drought, activation of ninja 6 and coronatine-insensitive 1a-like in K2 under drought+AMF, and strong reduction of LCR69 precursor in both K2 and KH. Within BR signaling, LOC100272702 and Zinc finger CCCH 46 were specifically expressed in K1, whereas K2 showed low expression of TIFY11e and ATP synthase D chain, with AMF reducing 14-3-3-like proteins. In auxin-related genes, Cytochrome P450 71A1 was enhanced in K1 under drought, while indole-3-acetaldehyde oxidase and pyruvate decarboxylase 2 were active in K2 under drought+AMF. Hybrid-specific regulation was observed for ARF22, ABP5 and SKU5-like oxidase, while reproductive development was supported by AMF-induced expression of CONSTANS-like 3, CONSTANS-interacting protein 6, knotted1 induced 1 precursor, tasselseed-2 and MADS19.
