# Supplementary material for: Genotype-specific responses of maize plants to Funneliformis mosseae under drought stress: phenomic and transcriptomic insights
Source: Front Plant Sci. 2026 Jan 6;16:1723031. doi: 10.3389/fpls.2025.1723031 (PMC12816378; doi:10.3389/fpls.2025.1723031)
Supplement: Supplementary file 2 [file Table1.docx]

***Supplementary Material***

| **Genotype** | **Watering** | **Mycorrhizal inoculation** | **Ear%** | **Tassel%** |  |
| --- | --- | --- | --- | --- | --- |
|  |  |  |  |  |  |
| **K1** | **60% FC** | **-** | 0 | 100 |  |
|  |  | **+** | 28.6 | 100 |  |
|  | **30% FC** | **-** | 0 | 62.5 |  |
|  |  | **+** | 33.3 | 100 |  |
| **K2** | **60% FC** | **-** | 100 | 100 |  |
|  |  | **+** | 67.5 | 100 |  |
|  | **30% FC** | **-** | 0 | 0 |  |
|  |  | **+** | 0 | 66.7 |  |
| **KH** | **60% FC** | **-** | 100 | 100 |  |
|  |  | **+** | 100 | 100 |  |
|  | **30% FC** | **-** | 100 | 100 |  |
|  |  | **+** | 100 | 100 |  |

Supplementary Table 1. Effect of the *F. mosseae* colonization on the flowering time in the inbred lines and their hybrid with normal watering (60% FC) and drought stress (30% FC) observed at the end of analysis time. K1: drought-tolerant parent, K2: drought-sensitive parent, KH: K1xK2 hybrid.


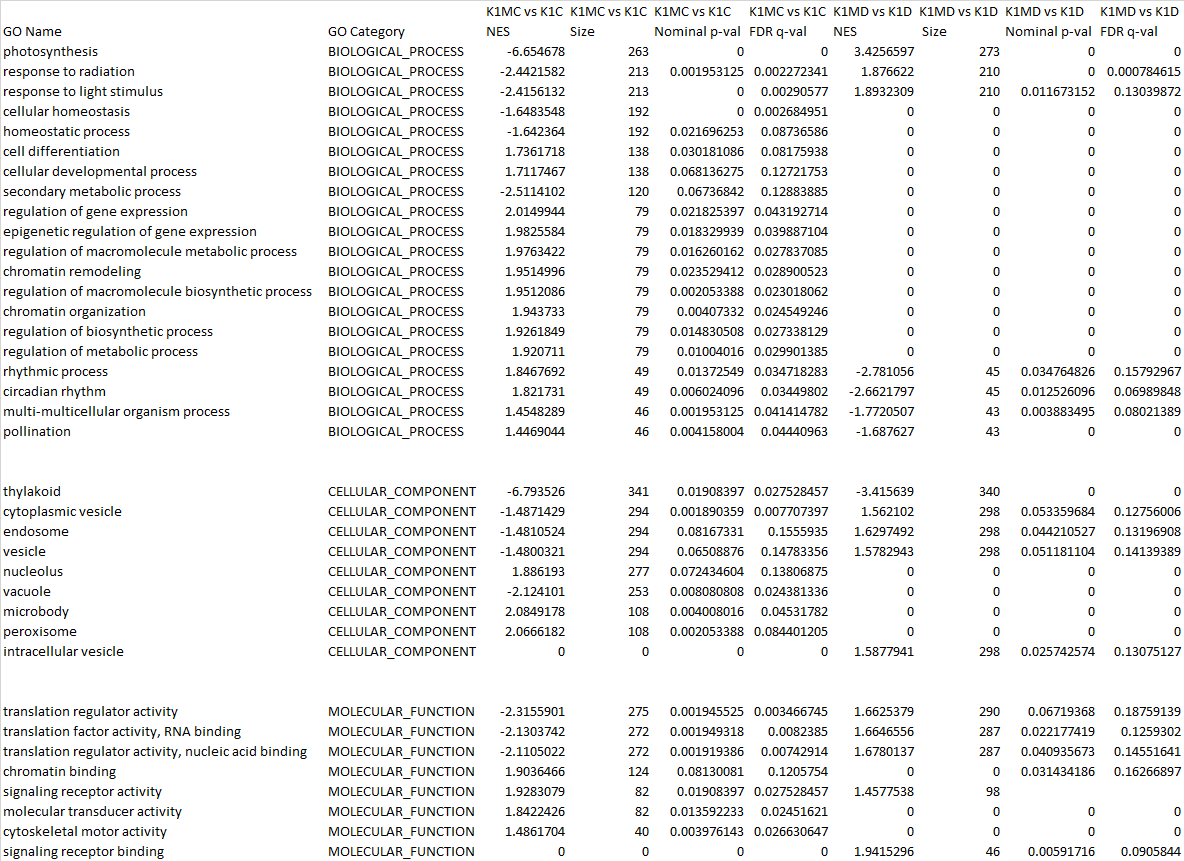


Supplementary Table 2. Statistics of GSEA analysis in K1:drought-tolerant parental line.

Supplementary Table 3. Statistics of GSEA analysis in K2:drought-sensitive parental line.


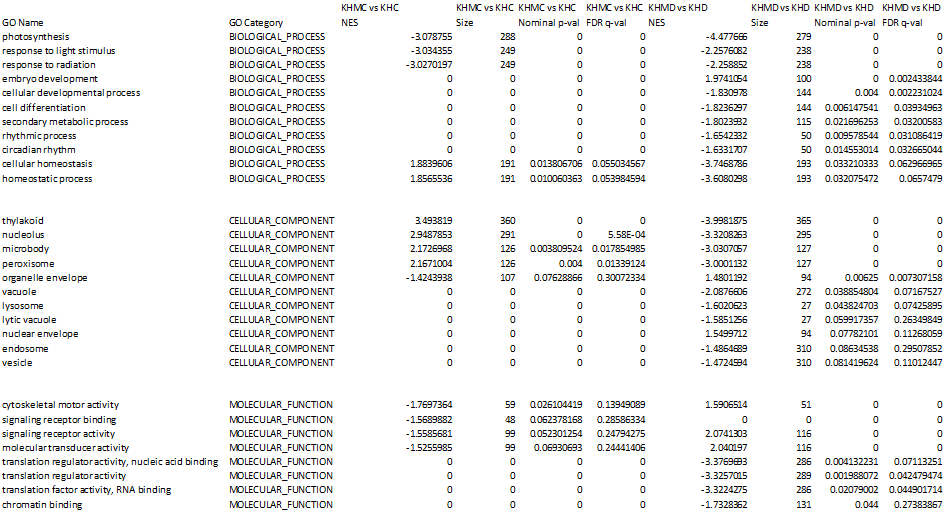


Supplementary Table 4. Statistics of GSEA analysis in KH:K1xK2 hybrid line.
